# Reservoir rock typing assessment in a coal-tight sand based heterogeneous geological formation through advanced AI methods

**DOI:** 10.1038/s41598-024-55250-y

**Published:** 2024-03-07

**Authors:** Umar Ashraf, Wanzhong Shi, Hucai Zhang, Aqsa Anees, Ren Jiang, Muhammad Ali, Hassan Nasir Mangi, Xiaonan Zhang

**Affiliations:** 1 Institute of International Rivers and Eco-Security, Yunnan University, Kunming, 650500 Yunnan China; 2 Institute for Ecological Research and Pollution Control of Plateau Lakes, School of Ecology and Environmental Science, Yunnan University, Kunming, 650500 Yunnan China; 3 Key Laboratory of Tectonics and Petroleum Resources, Ministry of Education, China University of Geosciences, Wuhan, 430074 Hubei China; 4 School of Earth Resources, China University of Geosciences, Wuhan, 430074 Hubei China; 5 Research Institute of Petroleum Exploration and Development, Petro-China Company Limited, Beijing, 100083 China; 6 Institute of Geophysics and Geomatics, China University of Geosciences, Wuhan, 430074 Hubei China; 7 School of Mines, China University of Mining and Technology, Xuzhou, 221116 Jiangsu China

**Keywords:** Solid Earth sciences, Geology, Geophysics

## Abstract

Geoscientists now identify coal layers using conventional well logs. Coal layer identification is the main technical difficulty in coalbed methane exploration and development. This research uses advanced quantile–quantile plot, self-organizing maps (SOM), k-means clustering, t-distributed stochastic neighbor embedding (t-SNE) and qualitative log curve assessment through three wells (X4, X5, X6) in complex geological formation to distinguish coal from tight sand and shale. Also, we identify the reservoir rock typing (RRT), gas-bearing and non-gas bearing potential zones. Results showed gamma-ray and resistivity logs are not reliable tools for coal identification. Further, coal layers highlighted high acoustic (AC) and neutron porosity (CNL), low density (DEN), low photoelectric, and low porosity values as compared to tight sand and shale. While, tight sand highlighted 5–10% porosity values. The SOM and clustering assessment provided the evidence of good-quality RRT for tight sand facies, whereas other clusters related to shale and coal showed poor-quality RRT. A t-SNE algorithm accurately distinguished coal and was used to make CNL and DEN plot that showed the presence of low-rank bituminous coal rank in study area. The presented strategy through conventional logs shall provide help to comprehend coal-tight sand lithofacies units for future mining.

## Introduction

**Figure 1:**
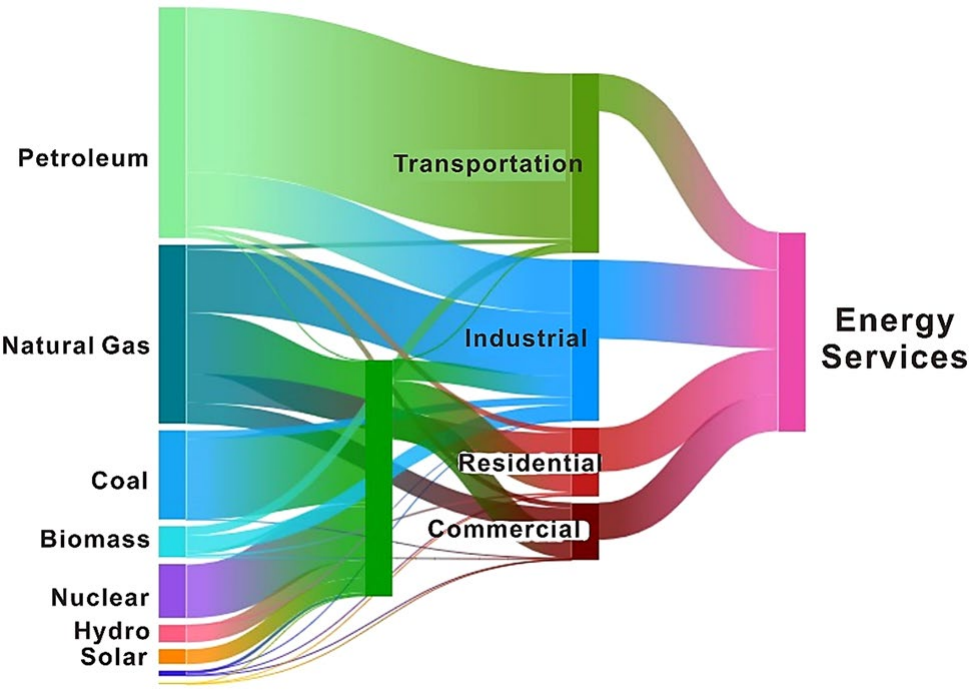
Natural gas and coal are the second and third most important energy sources for utilization of energy services.

Tight sand gas is the world's most important unconventional natural gas source for meeting energy needs and has become an important part of natural gas production^1^. Whereas, coal is a combustible sedimentary rock formed through breakdown of organic matter by the combined impacts of pressure, heat, and microbial activity over an extremely long period of time^2^. Authors^3^ illustrated, the world's top coal producers place a premium on research and development for coalbed methane (CBM). CBM exploration now places significant emphasis on the well logging technique used during the assessment of coal reservoirs. The development of the CBM cannot proceed without it since it is the cost-effective and dependable technique^4^. It is thus highly vital for CBM's resources exploration and development to enhance research on the accuracy and formation mechanism of CBM deposit for its exploitation to solve future energy crisis^5^. CBM formation is one solution to the problem of substituting unconventional gas for natural gas since it can overcome many energy services (Fig. 1). In the near and far future, coal will likely play a significant role in supplying needed energy. Therefore, the current research is focused on identifying the coal layers along with tight gas through advanced methods for utilization of energy services.

The identification of coal layer aids in the advancement of geological mapping by providing insights into the stratigraphy, sedimentary record, and complex structures of formations containing coal^6^. Coal is a very important and essential natural resource that is used for the purpose of power generation, steel manufacturing, and a wide range of industrial activities. Precise identification of coal strata is crucial for the identification of commercially feasible coal reserves^7^. Coal continues to be a substantial energy source globally, especially in nations that largely depend on fossil fuels for producing power. Precise identification of coal seams facilitates the effective extraction and usage of this energy resource^8^. An accurate comprehension of the distribution and properties of coal strata is essential for evaluating the environmental consequences linked to mining operations, such as land sinking, water contamination, and habitat devastation^9^. Precise coal layer identification data is an essential resource for governmental and regulatory bodies in the development of policies, land-use planning, and resource management strategies pertaining to the exploration, extraction, and conservation of coal^10^.

The technical difficulties associated with litho-typing and coal layer identification in geological formations stem from the complicated characteristics of coal deposits and the adjacent rock strata. Coal deposits often occur in formations that consist of a variety of rock types, including sandstone, shale, and limestone. This makes it difficult to precisely detect and separate coal seams from the surrounding layers of rock^11^. Coal is different from sandstone and shale in terms of coal quality parameters, such as ash content, carbon content, volatile content, and sulfur content^12^.

The evaluation of reservoir rock type for quality assessment is of utmost importance in the oil and gas business as it directly influences the productivity of the reservoir^13^. Therefore, well log analysis is crucial for estimating petrophysical parameters and it is performed to derive reservoir formation gas estimates from raw log data sets^14,15^. The use of artificial neural networks (ANN) and clustering approaches provide enhanced predictive accuracy compared to conventional and statistical models^16–20^. Different lithological groups may be identified using geophysical logs and petrophysical analysis, which can then be used as a guiding principle for future research^21,22^. Formation evaluation is still challenging to properly separate the lithological changes due to the complexity of the reservoir environment and the consequent diagenesis effectiveness^23,24^.

Prediction of subsurface lithofacies is vitally crucial for the future development and production of gas resources, as well as for the appraisal of possible future markets^25^. To evaluate lithofacies, many advanced tools are employed in oil and gas industry. The Self-Organizing Map (SOM) is a sophisticated algorithm used to detect distinct characteristics, such as facies, in data. It is particularly useful when core data is unavailable, facies data is limited, or when dealing with geoscientists that have less expertise^26^. K-means cluster analysis is a fast and reliable approach for clustering large datasets, which is easy to understand and often used to reveal significant patterns in large datasets^27^. A quantile–quantile (Q–Q) plot compares distribution shapes, showing how location, size, and skewness vary. Q–Q graphs compare data or theoretical distributions. Although less common, a Q–Q plot is more diagnostic than comparing samples histograms^28^. A t-distributed stochastic neighbor embedding (t-SNE) is a nonlinear dimensionality reduction approach that reduce high-dimensional data to a two-dimensional space for categorizing cluster distribution^29^.

It is quite contemporary to conduct research on distinguishing the lithofacies in a complex geological formation using logging tools^30^. Deng et al.^31^ utilized conventional logging tools to distinguish coal beds from shale, limestone and sand in the Hancheng area, China. Authors^32^ applied automated workflow to distinguish coal from shale and sand layers for unconventional reservoir characterization. In another study, authors^33^ applied advanced constrained sparse-spike inversion algorithm to distinguish thin beds of coal from sand and shale. Wood and Cai^34^ provided maximum and minimum threshold values to distinguish coal from other rocks through conventional logging performances. Hussain et al.^35^ summarized the basic characteristics of SOM and k-mean clustering for the identification of favorable gas regions. Kwilosz et al.^36^ utilized cluster analysis for grouping of shale-gas rocks in terms of their hydrocarbon potential generation. In another study, authors employed cluster analysis to distinguish gas facies in a carbonate reservoir^13^. Al-Dujaili^37^ studied RRT and storage capability of a carbonate rock in an oil field.

**Figure 2:**
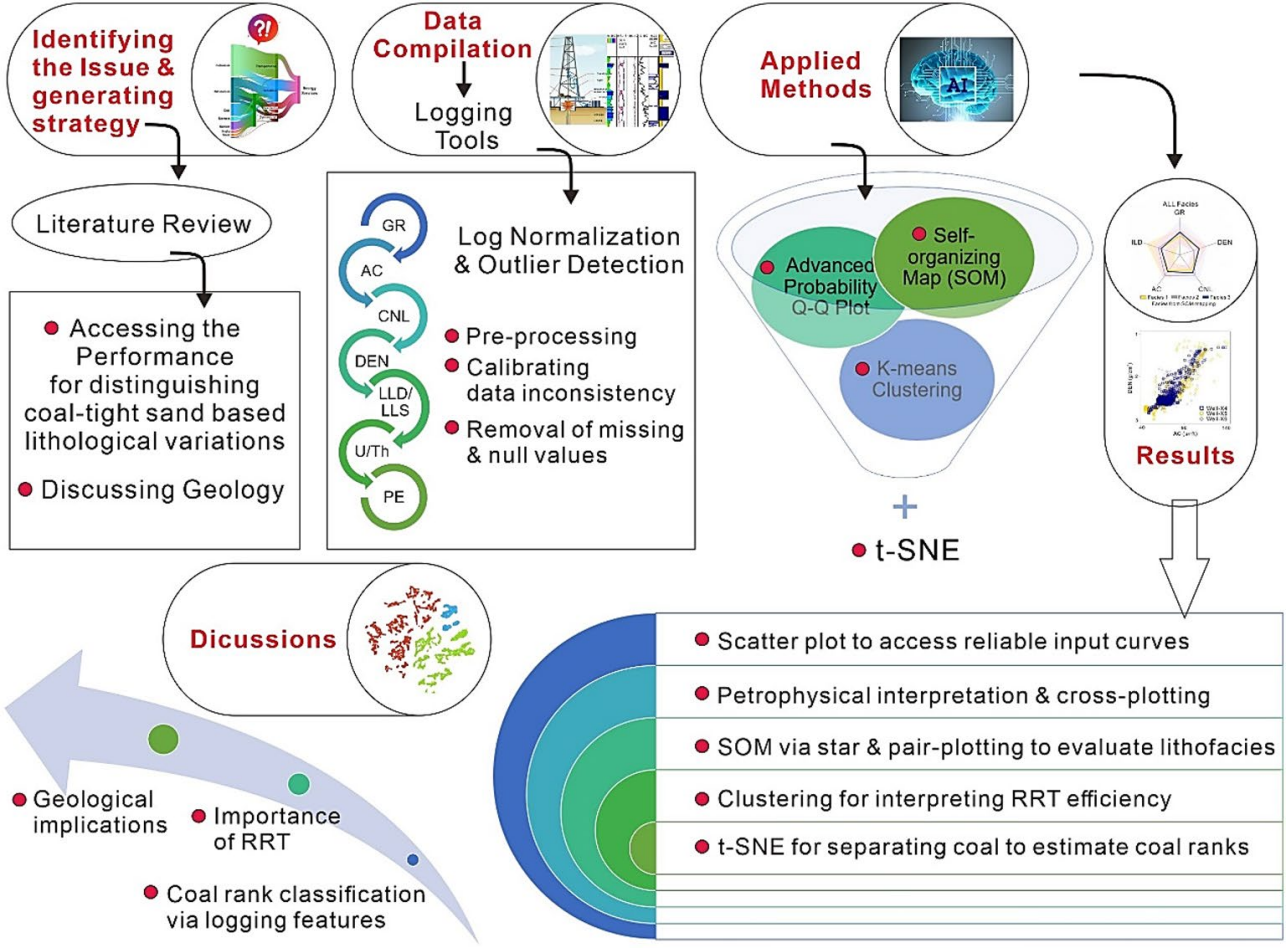
Workflow used to accomplish the research for RRT.

The purpose of the study is two-fold; a) to provide a workflow through advanced ANN and clustering methods to distinguish coal layer from tight sand and shale lithofacies; b) identify the quality of the reservoir rock type (RRT) and gas-bearing potential in a complex geological formation. To accomplish the study, three wells (Well-X4, Well-X5, and Well-X6) are utilized from a gas field in Ordos Basin, China. To accomplish the study, a reservoir thickness of about 150 m along with multiple conventional geophysical logs such as gamma-ray (GR), deep resistivity (LLD), shallow resistivity (LLS), acoustic (AC), density (DEN), neutron porosity (CNL), photoelectric (PE), thorium, (Th), and potassium (K) logs are employed. A literature is presented to show the performance characteristics and ranges of lithological variations prior to interpretation. Initially, input log curves log are conditioned to enhance the quality, and then AC log is used in Q–Q plot to distinguish the different distribution sets. Afterwards, petrophysical interpretation is performed to interpret the vertical extents of lithological variations. Multicrossplots, pairplots and histograms are drawn for a reliable distinction of coal with other lithofacies. An ANN based SOM is employed to visually analyze the lateral and vertical distribution of different facies in terms of distinct color codes. Also, K-means clustering approach is conducted to identify the quality of RRT for the distinguished lithofacies. An integration of SOM and clustering is presented for a comparison to identify the gas-bearing potential of RRT. In addition, we employed t-distributed stochastic neighbor embedding (t-SNE) algorithm to distinguish three distribution clusters to evaluate the coal ranks in the study area. At the end, the geological implications and importance of proposed workflow is presented. The presented workflow to accomplish our research is shown in Fig. 2.

The motivation of this project is to create an advanced workflow for accurately classifying reservoir rocks in intricate and diverse geological formations. This project aims to fill the following significant research gaps:

1. K-means clustering and SOM techniques are often used in the analysis of sand-shale sedimentary rock types. However, there is a scarcity of research for distinguishing coal from tight sand and shale through employed methods.
2. Utilization of advanced statistical tools, such as Q–Q plot and t-SNE, for effectively distinguishing the coal-tight sand facies in complex environments.
3. Determining the characteristics of the RRT and assessing the gas-bearing potential in a geologically heterogeneous formation.
4. Enhance the applicability of logging tools for the identification of coal ranks and coal versus non-coal zones.

### Geological characteristics of the study area

The research area is located in the Hangjinqi region, which is situated in the Yimeng Uplift and the northern Yishan slope and covers a vast area inside the northern Ordos Basin^38^. The petroleum play in the Hangjinqi region is as follows: the upper Paleozoic clastic rocks are the primary targets for natural gas development in the Hangjinqi region^39^. The predominant gas source rocks consist of thick beds of black mudstone and CBM deposited in the tidal flat delta lake environment, deposited in the Shanxi and Taiyuan formations^38^. The primary rocks that create the seal are the thick-bedded mudstones found in the upper Shihezi and Shiqianfeng formations^33,38,40^. The lower Shihezi formation is serving as the main reservoir in the study region and mostly consists of tight sands, including sandy fluvial conglomerate and sands with varying grain sizes^39,41^. The lower Shihezi formation consists of three sub-members known as member-1, member-2, and member-3, as described in the literature^42,43^. In a recent study, authors have shown that the member-1 of the lower Shihezi formation has favorable reservoir characteristics and has the capacity to support the extraction of gas reserves on a large-scale, both regionally and commercially^42^. Furthermore, the northern area of member-2 and member-3 of the lower Shihezi formation has shown promising zones for gas exploration^44^. In another recent study, authors revealed through sedimentary facies mapping that tight sand was deposited in braided channels and bars with maximum sand-ratio, mudstones were deposited in floodplains, whereas CBM showed deposition in swamp settings with lowest sand-ratio and lowest acoustic impedance values^40^.

## Performance of logging tools for distinguishing coal-tight sand based lithological variations

The natural radiations emitted from the minerals within the geological formations are quite beneficial in detecting the lithological variations through GR log. Potassium (K-40) associated with clay minerals is often the primary source of radioactivity in rocks, and it is hence more abundant in siltstones and mudstones. The uncontaminated sand and coal exhibit less GR values^45^. Usually coal shows low GR response, however, presence of uranium (U) series isotope concentration in coal shows higher GR response, while low values or absence of K-40 or Th series isotope will show low GR response^46^. Authors^47^ found that anthracite coals had GR values between 10 and 30 API, bituminous between 20 and 45 API, sub-bituminous around 20, and lignite from 0 to 25 API. However, presence of U or Th series within the coal shows high GR. On the other hand, sand shows lowest GR and shale shows highest GR readings^48^.

But, in addition to GR, other logs such as AC, DEN, CNL, and LLD readings are quite necessary for a reliable interpretation. To understand how lithology and porosity affect AC, an increase in porosity may be seen as the cause of a fall in velocity (increase of transit time)^49^. Because AC records velocity variations so precisely, therefore coal typically shows lower velocity (high AC) than other sedimentary rock types. The variation of AC values among distinct coal grades ranges from 110 to 155 μs/ft between lignite and anthracite^36^.

The ash content, grain density, and porosity, are important tools to interpret the DEN log behavior in sedimentary rocks^50^. Coal shows low DEN values ranges between 0.7 and 1.8 g/cm^3^, 2.2–2.5 g/cm^3^ for shale and clay, 2.5–2.65 g/cm^3^ for sand, and 2.7–2.9 g/cm^3^ for limestone^50^. Whereas, the DEN values in anthracite ranges from 1.4–1.8 g/cm^3^, 0.7–1.5 g/cm^3^ in lignite, and 1.2–1.5 g/cm^3^ for bituminous coal^47^. Thus, DEN is a powerful tool in detecting the coal strata as well as other subtle changes in rock densities and should also be used in conjunction to other logs.

**Figure 3:**
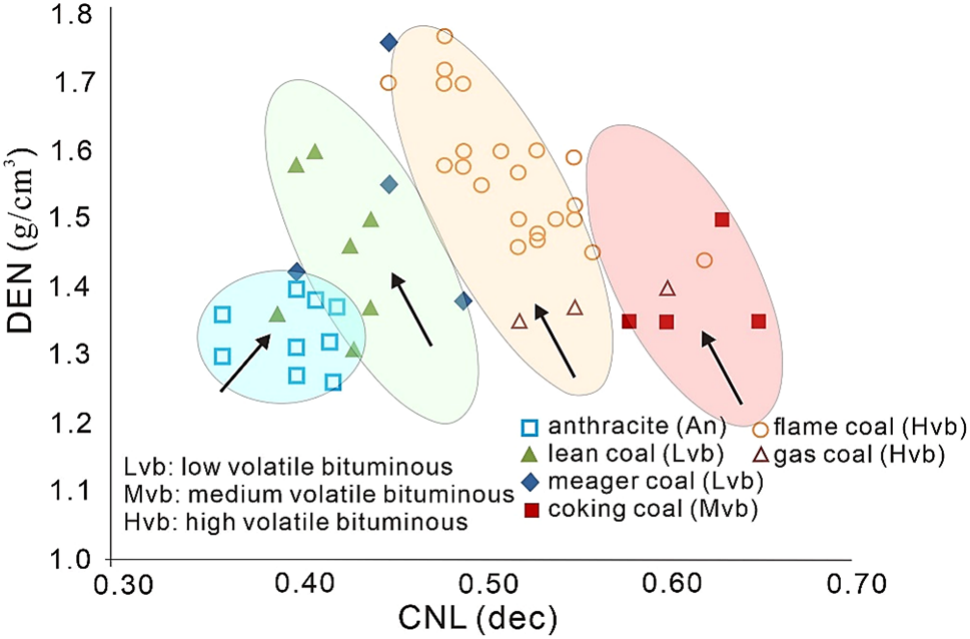
The log responses of CNL and density DEN in coalbeds with varying ranks^54^.

CNL, also known as porosity index, is sensitive to the volatile content of coals, particularly the hydrogen component^51^. It may be used as an estimation of coal volatiles. However, its measurement accuracy is influenced by the presence of non-hydrocarbon gases, namely carbon monoxide (CO) and carbon dioxide (CO_2_), which are often found in many coals^52^. CNL shows a high reading next to permeable fluid-filled rocks due to their high hydrogen concentration, but it also has a high reading next to a coal layer due to its high carbon content^53^. Clay with a high moisture content will likewise have a high CNL curve measurement. The generalized behavior of DEN and CNL logs towards coal with different ranks is shown in Fig. 3.

A substantial deviation in the resistivity curve is often seen directly opposite a coal seams because coal beds are much more resistive to the passage of an electric current than the rocks around them. When compared to other rocks, shale has a low resistivity, but coal and sand both have a high resistivity and may be mistaken for coal^49^. Therefore, it is challenging to dissociate the coal to sands based on the resistivity alone. Authors^47^ reported that the resistivity range for lignite is 2–10,000 Ω-m, 50–200 Ω m for bituminous coal, and for anthracite it is 2–8 Ω m. Bituminous, however, shows increased resistivity, whereas anthracite and lignite may exhibit low values. Therefore, owing to its wide range of variance, resistivity requires careful application.

In general, while differences in GR and resistivity across coal seams make them less trustworthy symbols, the lower DEN and higher CNL along with AC of coal seams make them reliable to identify^31^. The ranges of different logs to interpret the coal rank is shown in Table 1. The minimum and maximum well-log threshold values considered appropriate for differentiating coal strata in most exploratory boreholes conducted in the United States and China are provided in Table 2.

**Table 1:** Standard for detecting coal using well log ranges^31,55^ and are used in research screening to find coal^34^.

Coal rank	Matrix density (ρ_ma_) (gcm^−3^)	Bulk density (ρ_b_) (gcm^−3^)	Acoustic time (AC) (μs/ft)	Neutron porosity (CNL) (v/v)
Anthracite	1.5	1.3–1.9	80–90	0.35–0.45
Lignite	1.1	0.5–1.22	140–180	0.45–0.55
Immature bituminous	1.25	1.22–2.00	110–140	0.55–0.60
Mature bituminous	1.35	1.22–2.00	95–110	≥ 0.60

**Table 2:** Minimum and maximum threshold values of different logs to distinguish coal (modified after^34,54^).

Logs	DEN (g/cm^3^)	CNL (dec)	AC (us/ft)	LLD (Ω m)	GR (API)
Minimum ranges	1.1	0.35	90	10	10
Maximum ranges	1.95	0.75	160	2000	~ 100*

*GR varies depending upon the presence of Th or U isotope series.

## Methods

### Log curve normalization and outlier detection

Log conditioning is the preliminary step prior to interpretation and is applied on raw logs across zone of interest to enhance the quality of the log data^56^. Washout intervals have an effect on input log curves in the study region. As a consequence, log curves are dampened and skipped through. Therefore, logs conditioning plays a crucial function in strengthening the quality of the input log curves. Almost every clustering and classification procedure uses normalization to rectify log traces to avoid repeating errors^57^. Normalization and standardization stand out among data preprocessing and scaling approaches. Data is standardized by removing the mean and scaling it to unit variance. According to Ioffe and Szegedy^58^, the most popular normalization approach is to transform each sample to a range of zeros and ones using the lowest and highest feature values. Several prominent scaling approaches are hampered by dataset outliers. In this study, we applied the robust z-score scaling approach to overcome the outlier issue. This method involves removing the median and scaling the data according to the interquartile range, so according to Eq. 1:

1 $$z = \frac{x - \mu }{\sigma }$$

where $\mu = \frac{1}{N} \sum\nolimits_{i = 1}^{N} {\left( {x_{i} } \right)}$ shows the average value of the feature curve, *N* shows number of samples of feature curve, *x*_*i*_ provide value at ith sample point of a feature curve, *σ* is the standard deviation, and $\sigma = \sqrt{\frac{1}{N}} \sum\nolimits_{i = 1}^{N} {\left( {x_{i} - \mu } \right)^{2} }$.

### Quantile–quantile (Q–Q) plot

A Q–Q plot is an advanced probability algorithm that compares the quantiles of two probability distributions^28^. One of the quantiles of the second distribution (y-coordinate) is shown against the identical quantile of the first distribution (x-coordinate) at a given position (x, y) on the plot. This establishes the bounds of a parametric curve, where the index of the quantile range is the parameter.

A Q–Q plot compares distribution shapes, showing how location, size, and skewness vary. Q–Q graphs compare data or theoretical distributions. Although less common, a Q–Q plot is more diagnostic than comparing samples' histograms. Data sets and theoretical models are often compared using Q–Q graphs. This may offer a graphical goodness-of-fit rating rather than a summary statistic. Q–Q plots are useful for comparing distributions; unlike scatter plots, they do not need the values to be seen in pairs, nor do they require that the number of values in each group be the same^59^.

### Self-oganizing mapping (SOM)

The SOM is a mathematical method for classifying information into manageable portions for mapping through neural computing clustering technique to find hidden patterns in large datasets^60^. For SOM, the whole dataset's nodes are built via a training method. Random weights are put in each node to start training. Data is fed into the map after setup. The SOM can be summarized in to an Eq. 2;

2 $$W_{v} \left( {s + 1} \right) = W_{v} \left( s \right) +\uptheta \left( {{\text{u}},{\text{v}},{\text{s}}} \right)* \upalpha \left( {\text{s}} \right)*\left[ {{\text{D}}\left( {\text{t}} \right) - W_{v} \left( {\text{s}} \right)} \right]$$

where *s* stands for current iteration, *t* stands for index of targeted input data vector, *D(t)* denotes vector of targeted input data, *v* for node index on map, *W*_*v*_ shows current weight vector in the node, *u,v* exhibit the best matching units index on map, *α* is the learning restraint on account of iteration progress, and *θ*(*u*, *v*, *s*) illustrates the restraint owing to the distance from best matching units (BMUs).

A SOM model is created and calibrated in this investigation. It was developed to decipher the tight gas field's well logging calculations in order to construct the lithological section and identify the various lithofacies. The use of quantization error may serve as an indicator for assessing the quality of a SOM. The SOM consists of two primary elements that is composed of training and mapping. In the training phase, a square grid of neurons is used to initialize the network's learning process. Each side is represented by a weight vector with the same size as the input vector, and the weights are first determined at random. The network is fed by random input patterns in an iterative process that moves from the high-dimensional input space to the low-dimensional feature space^60^.

### K-means clustering approach

The oil industry largely depends on the evaluation of reservoir rock types to effectively manage reservoir output^13^. An evaluation of RRT is conducted using a cluster analysis approach. It is recommended that clusters engage in coordinated activities at each step of the many processes included by this method. In this study, we assess the effectiveness of RRT in characterizing the lithofacies by the use of a cluster analysis. Various types of rock serve as reservoirs, each with its own distinct characteristics in terms of POR and capacity for gas storage^61,62^. Cluster analysis is applied through various approaches such as nearest neighbor, furthest neighbor and average. The employed cluster analysis in the study can be described through Euclidean distance method through following Eq. 3;

3 $$d\left( {x,y} \right) = \sqrt {\mathop \sum \limits_{j = 1}^{n} \left( {x_{j} - y_{j} } \right)^{2} }$$

whereas *x* and *y* denotes the points in the Euclidean n-space, *x*_*j*_ and *y*_*j*_ denotes the Euclidean vectors and *n* stands for n-space.

## Results

### Log conditioning

**Figure 4:**
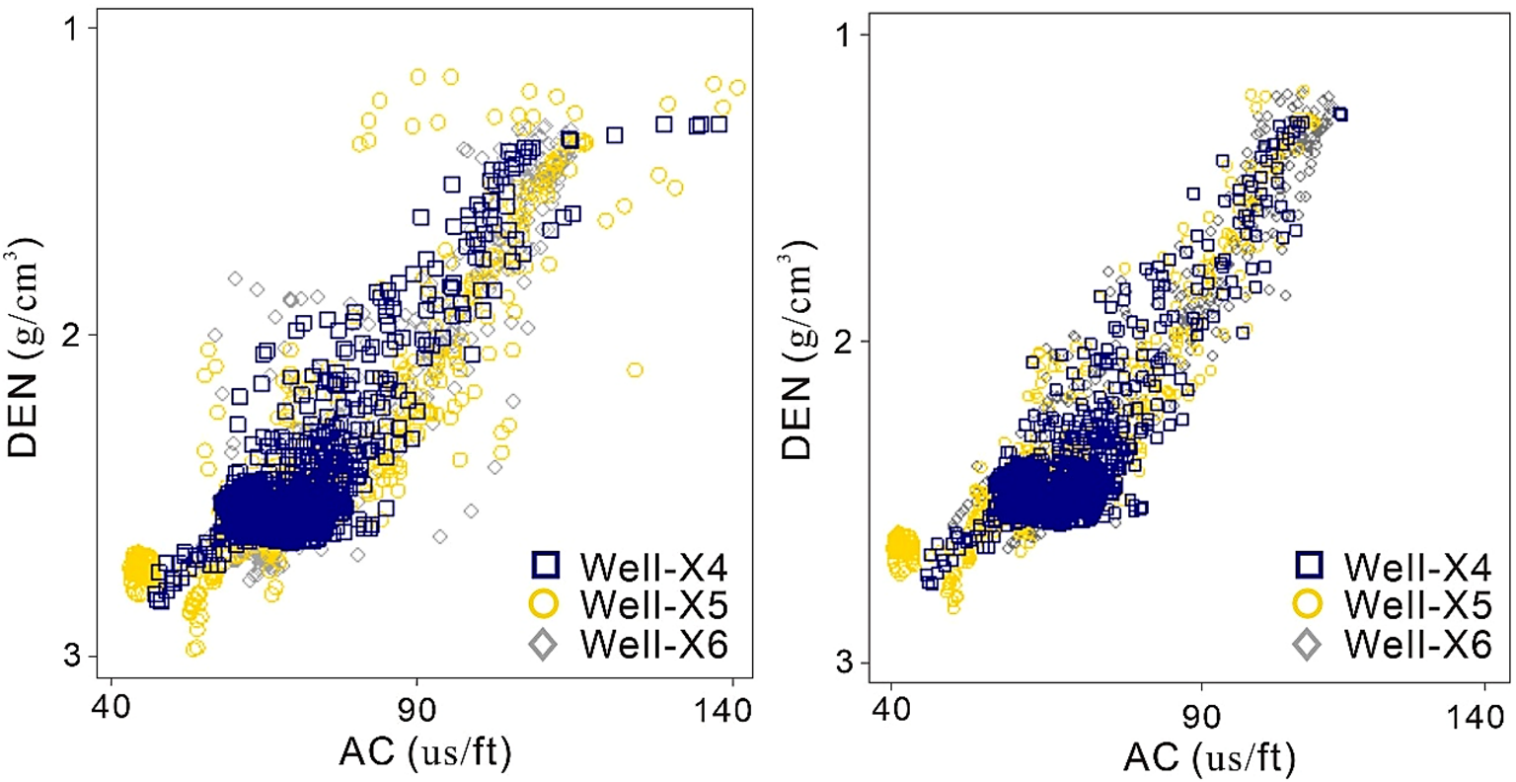
Normalization and removal of outlier. Left diagram shows multilwell AC and DEN log data before normalization, whereas right diagram shows after normalization.

The unprocessed data, distinguished by AC and DEN log curves, displays a dispersed arrangement of data points with notable outliers. Well-X5 has a higher level of variability in its input log curves when compared to Well-X4 and Well-X6. The use of normalization and outlier elimination methods led to notable enhancements, demonstrating strong correlations and clearly defined clusters within the multiwell dataset (Fig. 4).

It is important to highlight that all wells included in this research underwent conditioning using these approaches, particularly those with inadequate or missing data. By doing this rigorous preprocessing procedure, the data's trustworthiness and consistency are ensured prior to commencing any petrophysical investigations. The results of this first stage help to a more precise and significant interpretation of the petrophysical characteristics, hence improving the overall credibility of the region’s interpretation.

### Q–Q plot interpretation

**Figure 5:**
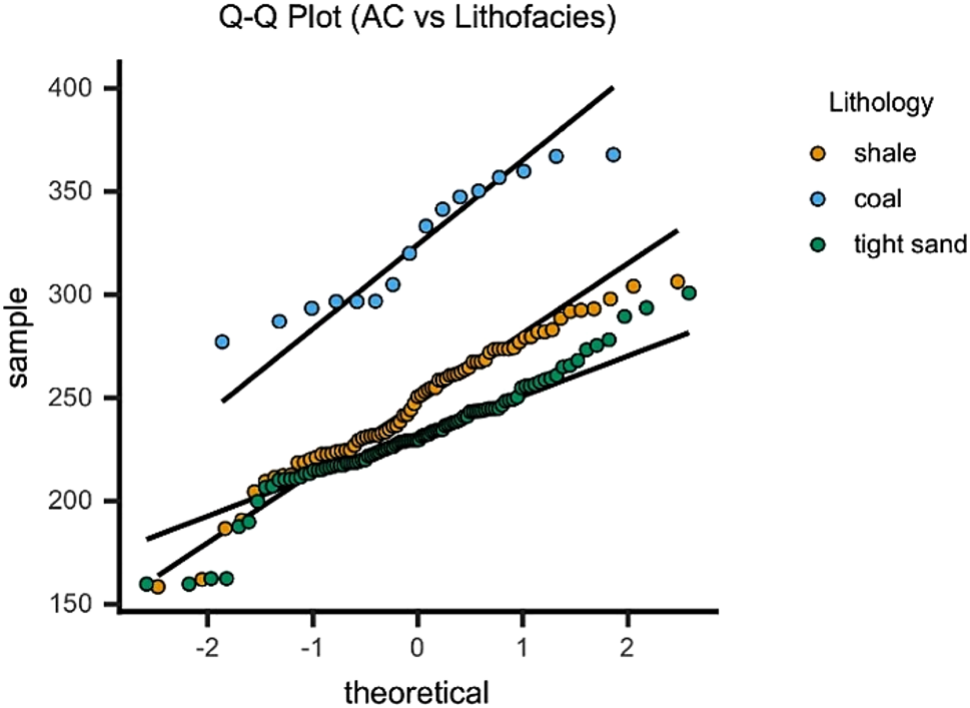
A Q–Q plot distinguishing the distribution sets through normalized AC (y-axis) and lithofacies (x-axis).

A Q–Q plots always show non-decreasing points from left to right for interpretation. The Q–Q plot follows 45° line y = x for identical distributions. In our case, a Q–Q-plot is imaged comparing the distributions of normalized AC log at average depths of different lithofacies. The curve pattern of various lithofacies suggests linear relationship for shale, coal and tight sand. The coal lies above the tight sand and shale illustrating that the AC values plotted on y-axis are high for coal (Fig. 5). In addition, the tight sand has low AC values as compare to shale lithofacies. The set of data for tight sand and shale are closely distributed to each other as compared to coal suggesting the closeness among these two sets. The closeness of these two distributions indicate the presence of frequent clay minerals in tight sand and shale. There are some outliers for all lithofacies suggesting complex geological formation and can be associated with the facies of variants of these lithofacies such as shaly coal, sandy shale, silt and clay.

### Logging-based lithological disparities

**Figure 6:**
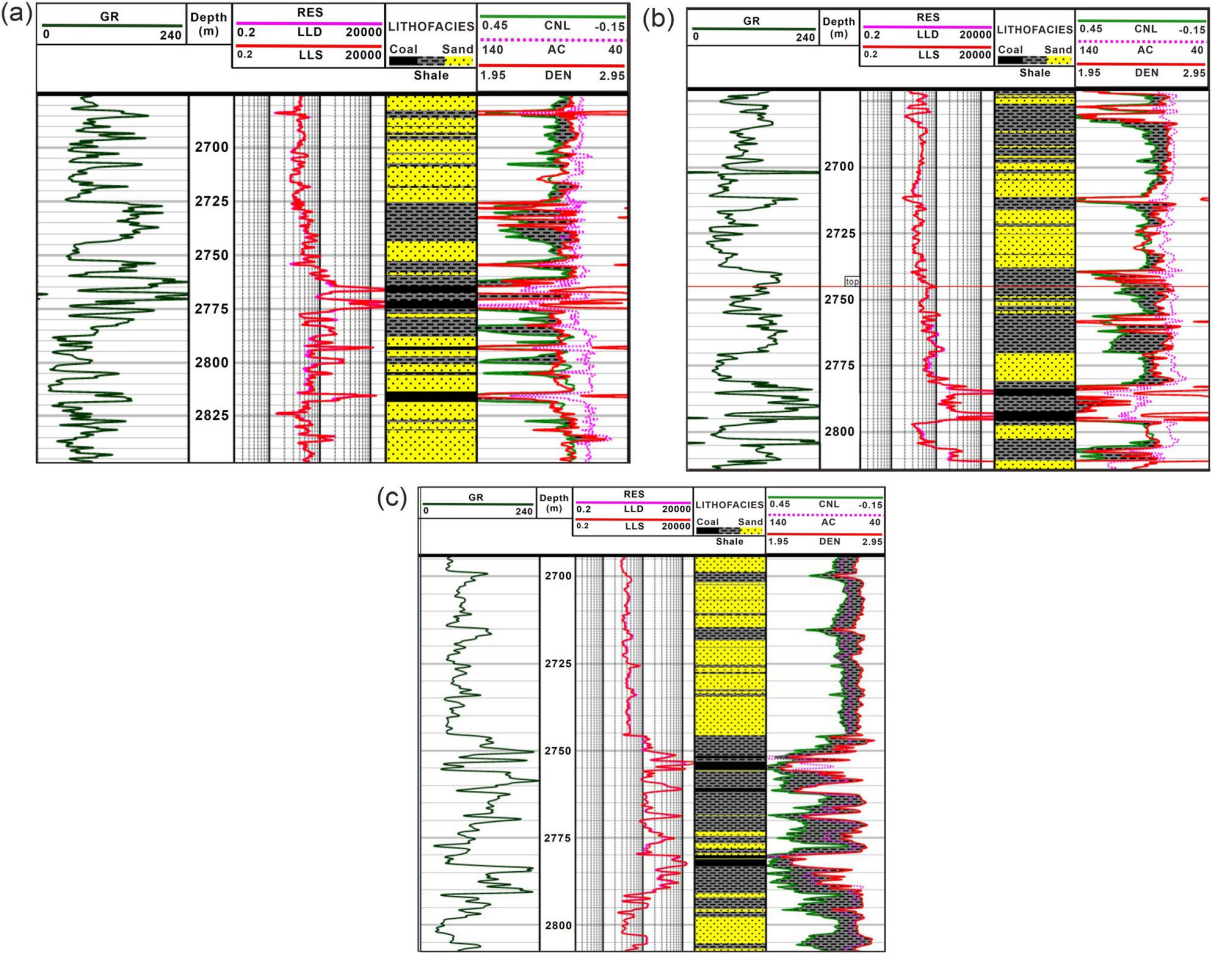
Petrophysical interpretation of Well-X4 (**a**), Well-X5 (**b**), and Well-X6 (**c**), showing the lithological disparities along with conventional log curves.

Different lithological variations are interpreted through combined analysis of AC, DEN, CNL, resistivity, and GR curves. The interpretation of conventional log curves within the designated research region facilitated the differentiation between tight sand, shale and coal strata. At the top of Well-X4 near 2765–2780 m, coal and shale facies are distinguished based on GR and resistivity logs (Fig. 6a). The coal layers show low GR, high LLD and LLS values, whereas shale facies show high GR and low resistivity values. However, based solely on the GR and resistivity logs, the coal layers are not easily distinguishable from tight sand due to their similar behavior of GR and LLD curves near 2795 m where an extremely thin coal layer sandwiched between tight sand show low GR and comparatively high resistivity values. In addition, a thin coal layer at 2805 m show exceedingly high GR and low resistivity values, which shows that GR and resistivity (LLD and LLS) logs alone are not reliable to interpret the coal strata. Therefore, GR and resistivity logs are used in conjunction with other conventional logs for a reliable interpretation. Coal and tight sand facies are reliably interpreted based on the fact that coal layer (~ 2815 m) provided low DEN, high CNL, and low POR values and shows distinct behavior compared to the tight sand that shows high DEN, and low CNL values.

Similar pattern is also observed for Well-X5 well log interpretation where two very thin coal layers close to 2785–2795 m show low DEN, high GR, moderate resistivity, high CNL and low POR values (Fig. 6b). These two thin coal layers are sandwiched between the shale layers showcasing the presence of shaly-coal facies intermixing with each other. The presence of clay close to a coal deposit giving rise an increased measurement of the CNL curve close to 2785–2795 m. On the other hand, tight sand close to 2800 m show low GR, moderate resistivity, high DEN, and low CNL values compare to coal and shale facies. Well-X6 shows high matrix values ranging from 2690 to 2750 m indicating the presence of tight sand permeable lithofacies. On the other hand, below 2750 m to 2790 m, thin coal layers are intermixed with shale lithofacies providing a possible seal for the above reservoir rock (Fig. 6c).

**Figure 7:**
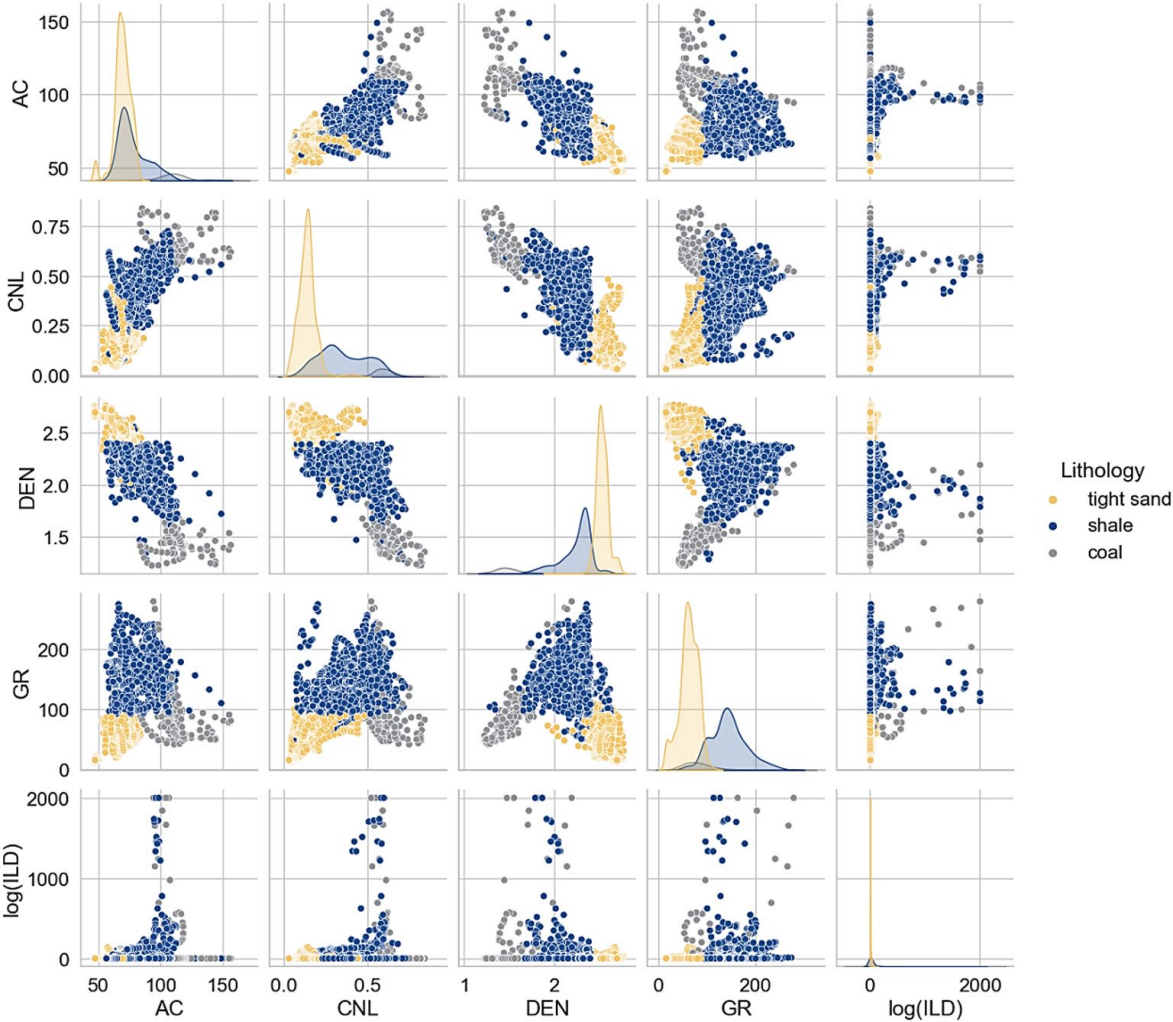
A scatter plot showing the lithofacies distribution using conventional logging tools.

The seaborn pairplot provided reliable distinction of shale, tight sand and coal. First row illuminates the behavior of AC log versus other logs illustrating that coal have the highest AC (90–150 us/ft), while tight sand has lowest AC and shale has moderate AC values. The second row illuminates the response of CNL log versus other logs which shows highest CNL for coal (≥ 0.45 dec), lowest for tight sand, and moderate values of CNL for shale facies. DEN log in the third row shows lowest values for coal (1.2–1.8 g/cm^3^), moderate for shale, and highest for the tight sand facies. In the fourth row, GR show lowest values for tight sand and highest for shale facies. However, GR shows broad values for coal with some data points exceed to 200 API. Whilst in the fifth row, the resistivity log shows poor clusters of coal along with tight sand and shale (Fig. 7). Henceforth, GR and resistivity logs, owing to their irregular patterns, making them less reliable for distinguishing coal from tight sand and shale in the study region.

### Crossplot analysis

**Figure 8:**
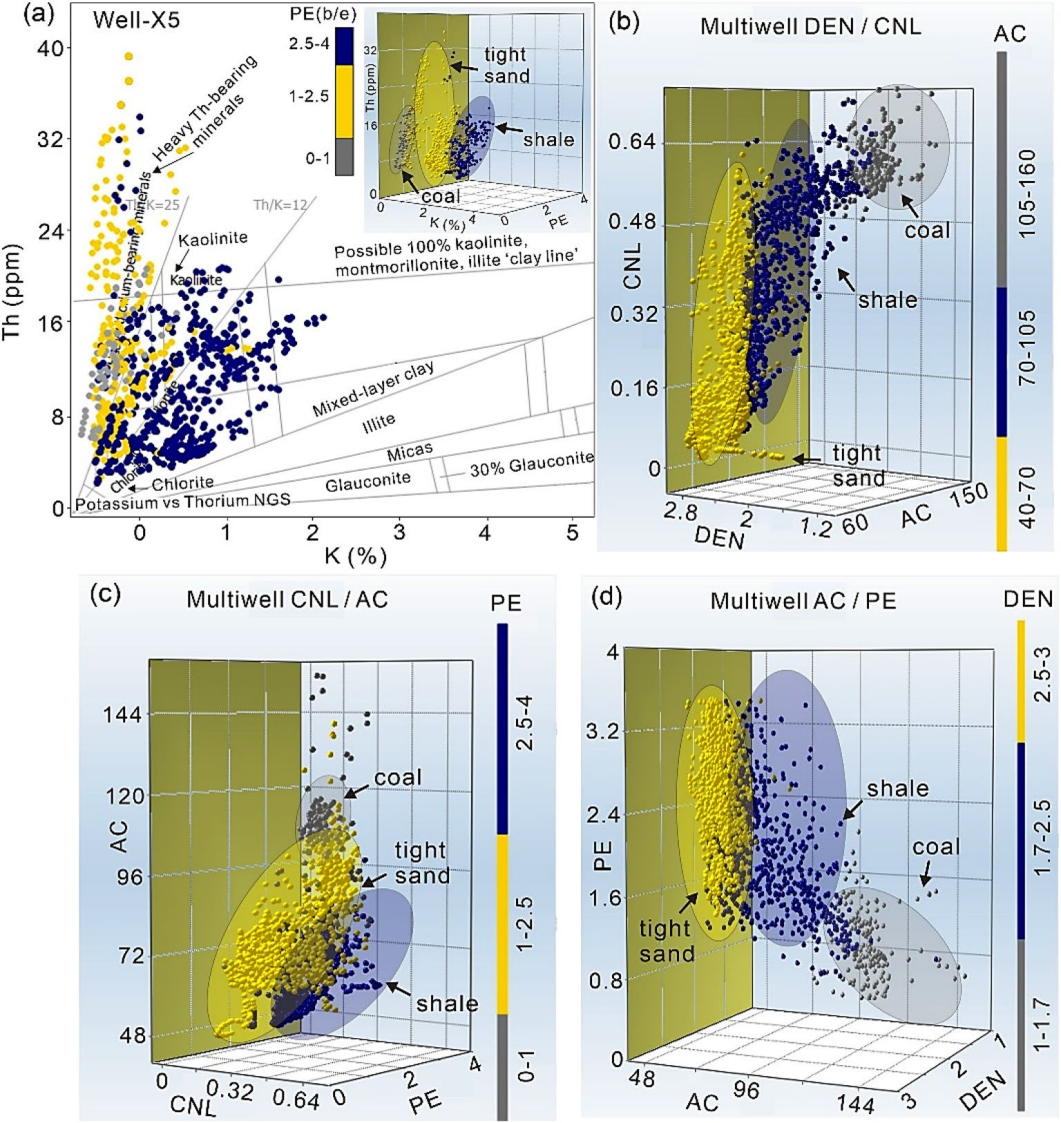
Crossplot analysis among conventional logs to distinguish coal-tight sand lithofacies. (**a**) K versus Th color coded with PE, (**b**) DEN versus CNL color coded with AC, (**c**) CNL versus AC coloring with PE, (**d**) AC versus PE with DEN z-axis values.

The occurrence of certain minerals with high GR values may contribute to the development of a diverse mineral composition in sand reservoirs. Hence, the use of certain crossplots, such as those using natural gamma-ray (NGR) spectroscopy logs (e.g., Th and K), proves to be very advantageous in the identification of minerals with elevated GR responses^63^. The PE log is also quite beneficial in identifying lithological variations. The PE value less than 1 shows coal, around 2 suggests sand, and values near 3 denotes shale and values close to 5 gives the presence of limestone^64^. Crossplot made for Well-X5 between K and Th logs color coded with PE log shows the presence of coal with quite low PE values. Clay content in sand may be determined by analyzing the Th value, whereas the presence of K indicates the concentration of k-feldspar and mica minerals^65^. Results shows that sand-shale intercalations are mostly comprised of mixed-clay layers including mostly kaolinite and minor illite-rich minerals, which causes overlapping of tight sand and shale lithofacies (Fig. 8a). In addition, gray colored coal lithofacies clustered in the heavy thorium-bearing minerals ranges resulted in higher GR values among the coal in the geological formation.

The multiwell plots of DEN versus CNL color coded with AC log (Fig. 8b), CNL versus AC color coded with PE log (Fig. 8c), and AC versus PE color coded with DEN log (Fig. 8d), reliably distinguished the coal-tight sand based lithofacies. Coal shows low DEN values ranges from 1.25 to 1.65 g/cm^3^, low PE values of less than 1 b/e, high CNL values close to 0.3–0.7 dec, and high AC values higher than 100 us/ft. On the other hand, tight sand showed high DEN values close to 2.60 g/cm^3^, moderate PE value of average 2 b/e, low CNL values mostly clustered around 0.2–0.3 dec, and low AC values (40–70 us/ft). Shale variants marked evidently with blue color show intermediate ranges between coal and tight sand.

### SOM-based lithofacies evaluation

**Figure 9:**
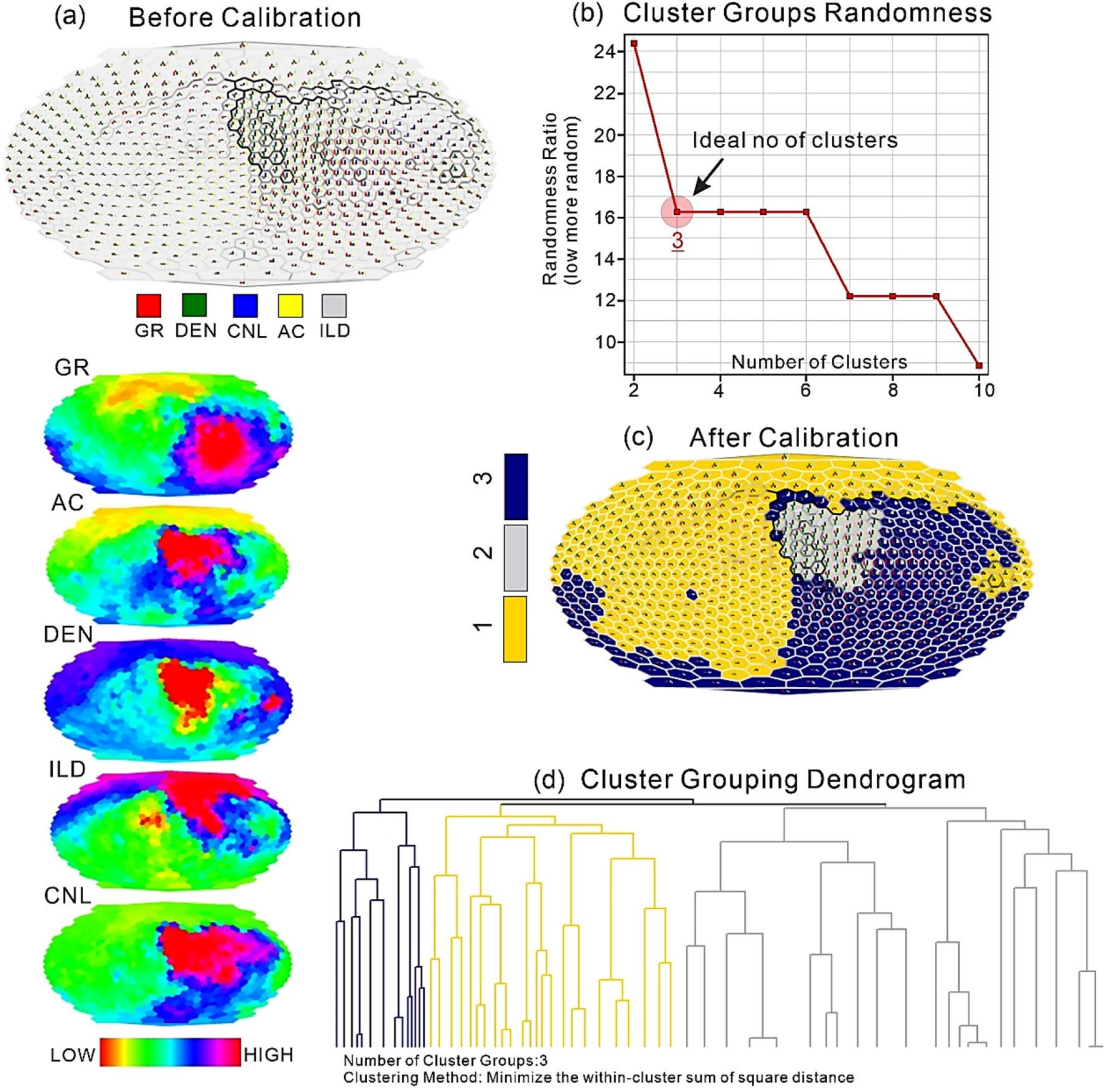
2D facies distribution through SOM are created using GR, CNL, AC, and DEN log data. A color index shows each log's contribution. (**a**) Dispersed maps of utilized logs before calibration. (**b**) Cluster randomization plot showing the ideal number of clusters. (**c**) SOM-clustering results after calibration. (**d**) A dendrogram showing the group of three identified clusters.

In the initial phase of the SOM modeling, a completely dispersed map of study wells is developed as seen in Fig. 9a. SOM training through five logs (GR, AC, DEN, ILD, and CNL) organizes input space to encode pattern lithofacies two-dimensionally. After training, the map topographically organizes, suggesting the network can map all input data into SOM feature space. Mapping aims to estimate the major rock types from well logs in terms of various color codes. The cluster randomization plot showed that 3 is an ideal number of cluster to use because of first sharp down turn introducing less randomness (Fig. 9b). Results of SOM model showed three significant lithofacies clustered as 1, 2, and 3 (Fig. 9c), and three cluster groups are shown in cluster grouping dendrogram (Fig. 9d). Each facies color code provided horizontal and vertical distribution function to describe the interpreted lithology of the multiwells.

Recognized facies in the reservoir region of around 150 m illustrate the subtle changes in lithological components in terms of distinct color codes to distinguish the three main facies such as tight sand, shale variants, and coal. Facies coded in gold color with assigned number 1 indicated tight sand, gray color with number 2 showed the presence of coal, whereas those in blue color with number 3 denoted shale, siltstone and associated clay minerals. The SOM model reliably validated the presence of hydrocarbons lithological variations in terms of both their magnitude and potential.

**Figure 10:**
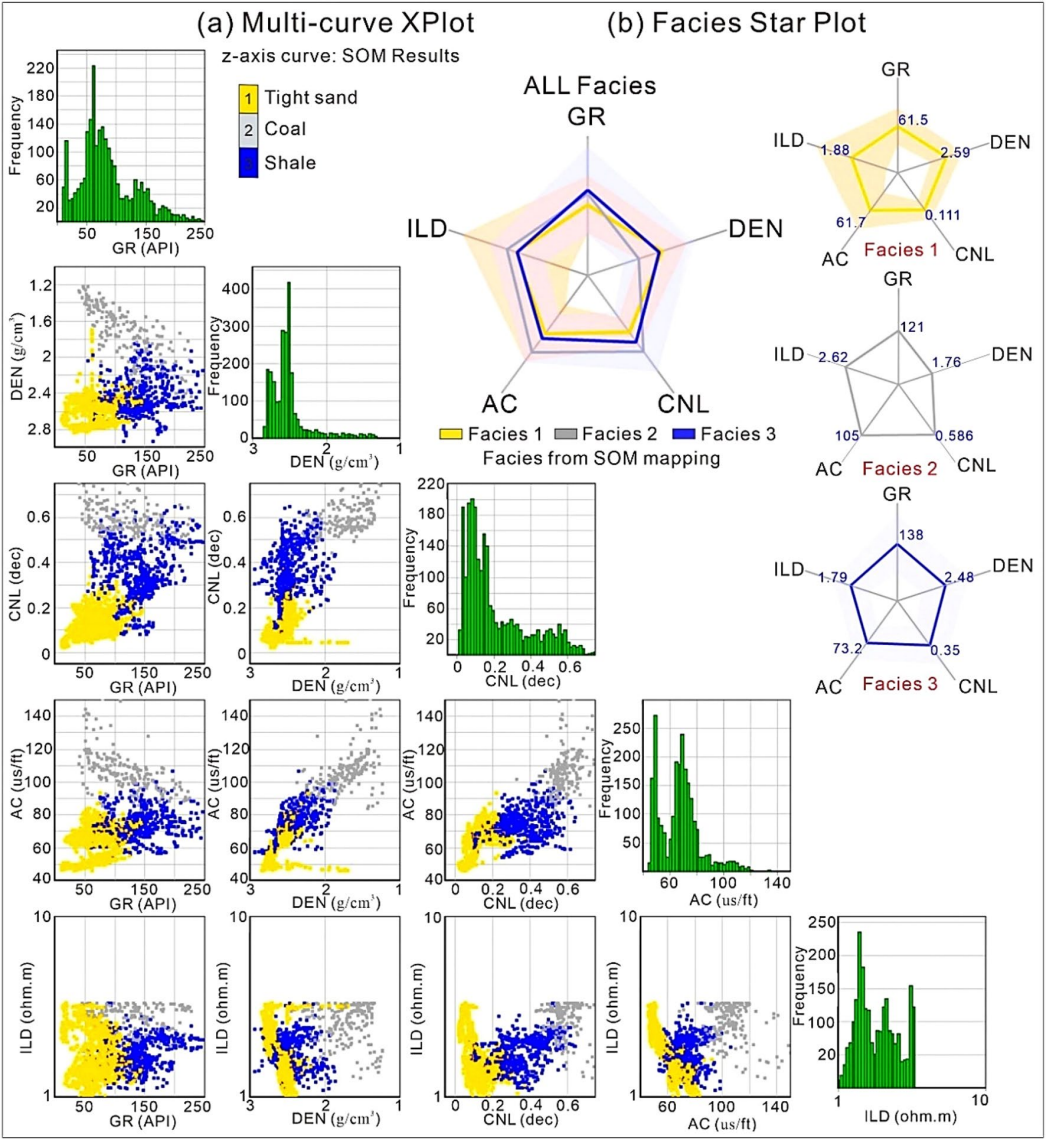
(**a**) SOM results displaying the multicurve crossplot and frequency histogram distributions via DEN, CNL, AC, GR, and ILD. (**b**) Star plot showing the facies generated from SOM mapping and the mean value of each log on three facies 1, 2, and 3.

Results of SOM analysis in the form of multicurve crossplot and starplot facies mapping is shown in Fig. 10 through input five log cuvres (DEN, CNL, AC, GR, and ILD) that provided a clear distinction of three types of clusters presented by three color codes (Fig. 10a). These three codes reliably show that code 1 facies cluster belong to tight sand gas-bearing facies since these facies show high DEN, low AC and CNL, and low GR values. Whereas, code 2 facies cluster presented with gray color represents coal facies having low DEN, high AC and CNL values. Whereas, facies 3 color coded with blue color denotes intercalations of shale/silt and clay minerals and signifies non-gas bearing lithofacies. On the other hand, the mean value of each log on all three clusters/facies are visualized by starplot pattern which provided the detailed presentation of each facies type in the reservoir geological formation (Fig. 10b). The mean values of each log type along with each facies is shown in Table 3.

**Table 3:** SOM results of star-plot facies mapping showing the mean values of each log type along with associated cluster/facies no.

Cluster/facies no	No. of data values	GR (API)	DEN (g/cm^3^)	CNL (dec)	AC (us/ft)	log (ILD)	Lithofacies Interpretation
		Mean	Mean	Mean	Mean	Mean	
Facies 1	677	53.791	2.6874	0.06766	50.653	2.5763	Tight gas
Facies 3	300	143.16	1.9344	0.56231	96.742	2.4601	Coal
Facies 3	1440	89.939	2.5141	0.20909	70.83	1.4985	Shale intercalations

### Clustering-based reservoir rock typing (RRT)

**Figure 11:**
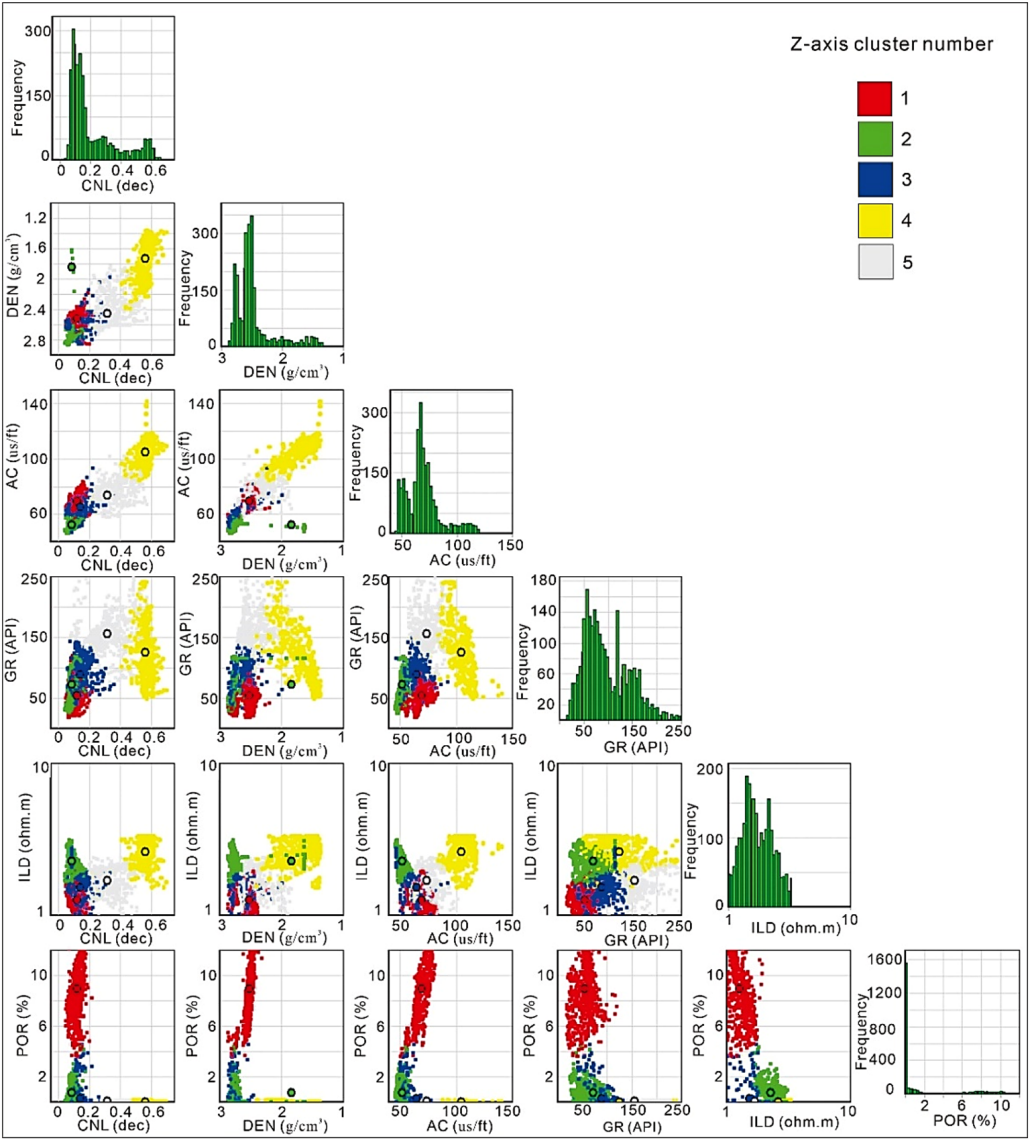
Results of k-means clustering approach showcasing 5 clusters using GR, AC, CNL, DEN, ILD, and POR logs.

The quality of RRT for the distinguished lithofacies is evaluated by the use of a k-means cluster analysis. The input data for cluster analysis consisted of the DEN, CNL, AC, GR, ILD, and POR logs. Five clusters are chosen to showcase all the differences in the data. The image depicting histograms and crossplots of the curves generated from the input data via k-means clustering method for rock type groups is shown in Fig. 11. The detailed results of each cluster type is shown in Table 4, which provides the mean values and statistical information for each five cluster based on the data used. The clustering-based RRT method shows changes in the lithofacies volume and petroleum potential. The gas-bearing lithofacies of tight sand are quite productive with good POR values, while those of coal and shale having silt and clay intercalations represents non-gas bearing lithofacies. Based on the results obtained from the cluster assessment, it has been determined that the cluster 1 exhibit promising characteristics for the reservoir zones within the designated research region and termed as tight gas reservoir rock type, whereas other clusters 2, 3, 4, and 5 provide the poor characteristics for the potential gas zones due to lowest porosity values, hence, termed as poor-quality RRT.

**Table 4:** The results of the cluster analysis performed on each rock type.

Cluster no	Cluster points	Cluster spread	CNL	DEN	AC	GR	ILD	POR	RRT interpretation
			Mean	Mean	Mean	Mean	Mean	Mean	
1	629	0.9168	0.12089	2.5189	69.161	54.691	1.2582	8.9058	Tight gas reservoir
2	488	0.7721	0.08938	1.839	51.972	71.966	2.2756	0.74089	Poor rock type
3	582	1.47	0.1399	2.71	65.198	88.918	1.5279	0.27405	Poor rock type
4	272	1.339	0.56593	1.7381	104.8	125.53	2.6167	0.01802	Poor rock type
5	614	1.05	0.31892	2.459	73.557	155.47	1.6908	0.09778	Poor rock type

**Figure 12:**
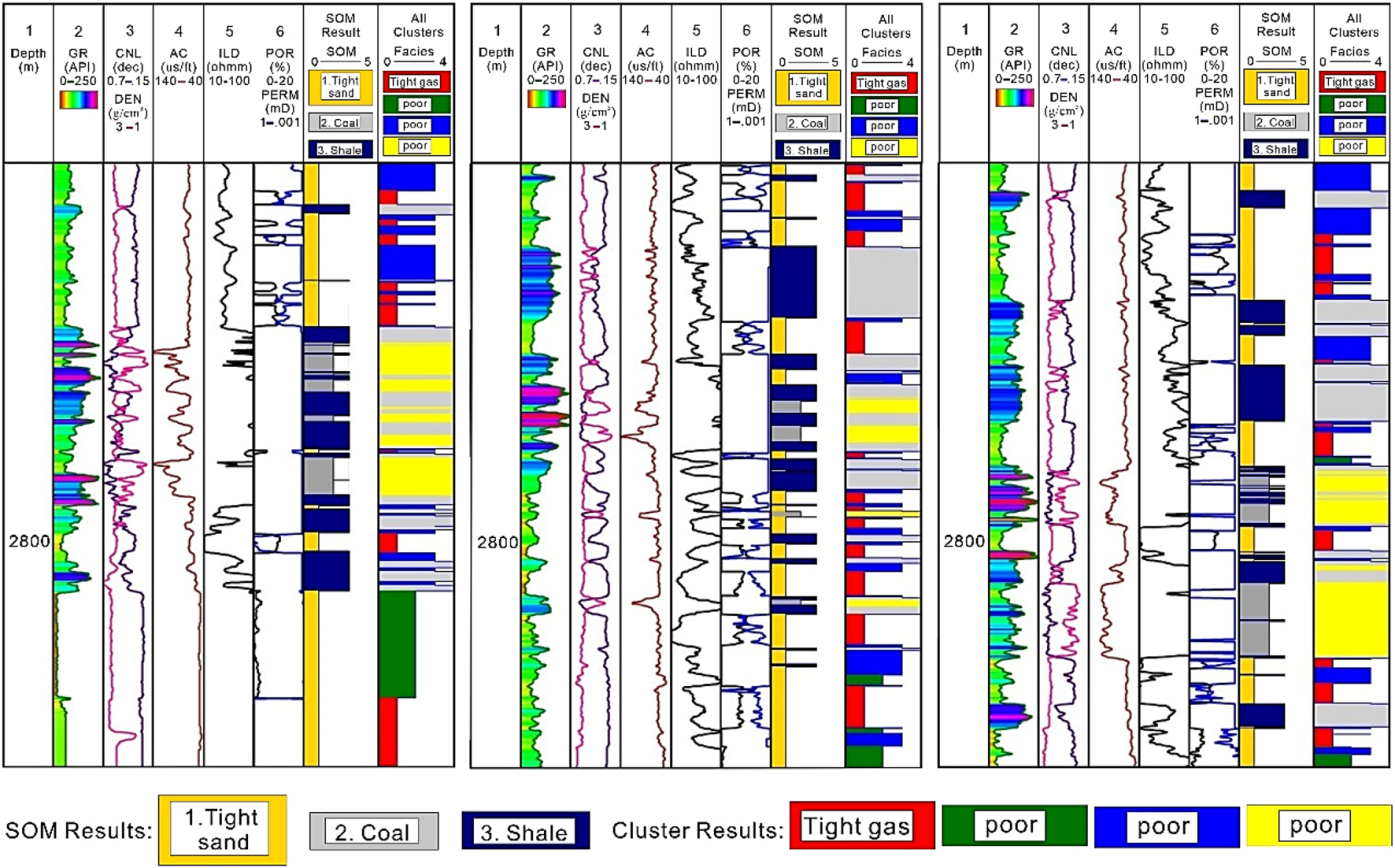
SOM and k-mean clustering assessment results along with input logs (GR, CNL, DEN, AC, ILD, POR) showing the facies types and RRT.

Figure 12 shows the integrated results of the SOM model and k-means clustering assessment indicating the existence of main lithofacies, type of RRT, and also sheds light on the size and hydrocarbon potential changes in the observable vertical lithofacies. The interpreted lithological description of the multiwells is built using the horizontal and vertical distribution functions, with a color code and an assigned number to each kind of facies. Tight sand facies color coded in gold color with assigned number 1 exhibit low GR, high DEN, low CNL, low AC, and highest POR values suggesting tight gas-bearing RRT as indicated by red colored cluster. Whereas, shale/silt facies color coded in blue color with assigned number 3 shows highest GR and lowest POR values providing the evidence of clay minerals in shale/silt facies and referred as non-gas bearing poor-quality RRT as shown in the last two corresponding columns with 2 (green colored), 3 (blue colored), and 5 (gray colored) clusters. On the other hand, coal lithofacies color coded in gray color with assigned number 2 shows highest AC and CNL, lowest DEN and POR values also indicative of a lithofacies with poor-quality RRT as indicated in the last column with corresponding clusters such as no 4 (yellow colored) and 5 (gray colored). The integration of these two methods (SOM and K-means clustering) offered a simple approach to evaluate the log capabilities in terms of discriminating between favorable and unfavorable facies within a set. Improved lithology interpretation and more consistent outcomes have resulted from combining the SOM method with multi-crossplots and k-means clustering assessment.

## Discussion

### Classification of coal rank through geophysical logging characteristics

Laboratory analysis is the most reliable way to determine coal composition^34^. However, number of studies have been published with some success to identify coal ranks and coal zones from non-coal zones^31,34,53,54,66^. Mao et al.^66^ combined the CBM records from six fields from the Ordos basin, Junggar basin, Songliao basin, and Qinshui basin, for the identification of coal rank using geophysical log characteristics that resulted in 95% accuracy when compared the results with laboratory analysis. The fundamental set of well logs may be used not only to differentiate coals from other rock types but also to accurately forecast their brittleness, fracability, coal rank, gas content and proximate analytical components with dependable precision^34^. In another study, a crossplot of DEN versus CNL logs was used to successfully find coal ranks in different CBM areas across several basins in China^54^.

**Figure 13:**
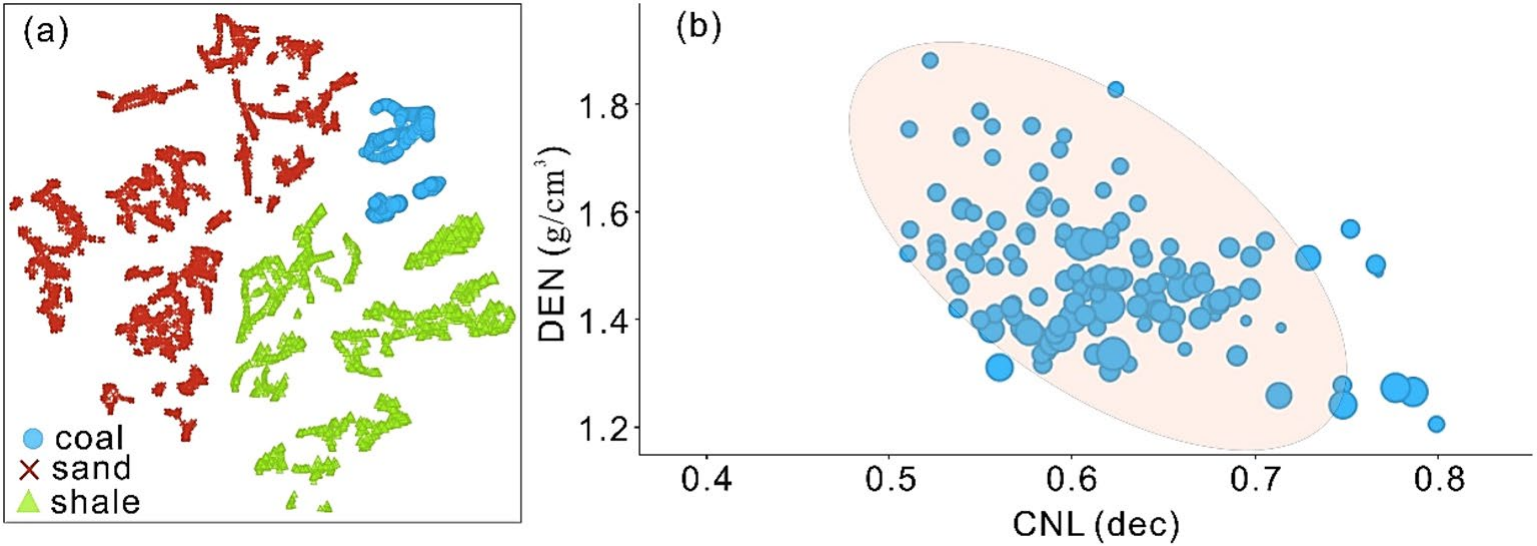
(**a**) t-SNE algorithm allowing easy visualization and interpretation of coal against tight sand and shale. (**b**) A DEN versus CNL crossplot to provide an insights of coal data records for interpreting the coal rank. AC log is used to plot the magnitude in terms of sizes.

We have utilized the DEN versus CNL plot presented by Zhao et al.^54^ to identify the coal rank in the study area. Initially, we have utilized advanced t-SNE algorithm to accurately distinguish the coal, shale and tight sand. In t-SNE, each high-dimensional item is represented by two-dimensional points, with comparable points being closer and dissimilar points being further apart^16^. Multiple metrics were used in the assessment of t-SNE. The perplexity value was set to 25, the exaggeration value was set to 4, standardization was performed before to applying the PCA components, and the results were visualized using zero jittering. Results of t-SNE algorithm categorized three unique clusters reliably distinguishing coal from tight sand and shale (Fig. 13a). We then selected and utilized these coal data point to plot a multiwell DEN versus CNL crossplot. AC log was also used in conjunction to visualize the magnitudes in terms of their sizes. Larger size shows maximum AC values, whereas lower size of circle shows minimum AC value. Coal shows thick clusters with high CNL values that ranges from 0.49–0.79 dec, and variable DEN values ranges from 1.2 to 1.8 g/cm^3^ (Fig. 13b). Considering Table 1 for classifying coal rank, the coal in the study area can be ranked as immature or high volatile bituminous. However, laboratory analysis will provide further insights to interpret the coal rank in the study area.

### Geological implications and importance of rock typing efficiency

In 2019, China used 64% (691 Mt) of the world's met coal, making it the biggest consumer^67^. China needs to find other sources of energy to make up for its energy shortage. Along with unconventional tight gas, coal is one of the most abundant fossil fuel in China and mainly used in the thermal power plants, iron and steel industry to meet the energy needs. Coal is expected to play an important part in meeting the short to long-term energy needs of China. Henceforth, accurately identifying CBM is quite essential and have regional implications inside China.

Hangjinqi was investigated for natural gas some 40 years ago, but efficient consideration began a decade ago for pure gas exploration. Although several wells have been drilled, study area still has many unknown productive zones^44^. Insufficient research has been conducted on the distribution of coal structure in the study region, which is one of the major causes for the identification of unknown gas potential zones. Thus, developing an advanced workflow utilizing advanced statistical clustering and classification techniques is essential for accurate distinction of lithofacies and rock types.

RRT provide important insights regarding the favorable gas-bearing regions, and reservoir quality potential. In addition, RRT conveys a distinct correlation among POR, PERM, storage capacity, and deliverability^61^. RRT efficiency also helps in understanding the recovery of reservoir fluid and oil^68^, and carbon capture and utilization storage^69^. In our study, we have employed SOM and Q–Q plot for the recognition of distinct lithofacies, and the gas-bearing efficiency was tested via RRT cluster analysis. Tight sand provided good POR and PERM values and can be targeted for unconventional natural gas exploitation and future drillings. The shale and coal showed distinct petrophysical characteristics as shown in Table 5, and were reliably distinguished through multicurve crossplot, facies star plot, lithological section and t-SNE.

**Table 5:** The estimated ranges of conventional logs for the different rock types in the study area.

Sedimentary rock type	RHOB (g/cm^3^)	GR (API)	AC (μs/ft)	CNL (dec)	log (ILD)	PE (b/e)
Coal	1.25–1.65	40–130	90–150	0.45–0.75	2–4	≤ 1
Shale intercalations	2.1–2.5	60–200	60–100	0.15–0.55	1–2	1–3
Tight sand	2.5–2.69	10–80	40–70	0–0.35	1–3.5	≥ 2–4

The proposed workflow has strong geological implications since the accurate distinction of coal and shale shall expand the geological considerations in terms of depositional environment and structural as well as stratigraphic variations. Also, it will play a crucial role in understanding the heterogeneity of coal formations, impacting saturation through variations in lithology and structural characteristics. Furthermore, the delineated tight sand gas-bearing potential will contribute to a more comprehensive geological framework for understanding saturation dynamics of Hangjinqi area. Nevertheless, the interpretation methodologies used in this work have broader value for the coal related complex geological formations. However, since variations exist across various geological environments and coal strata globally, a careful interpretation is needed. We disclosed that DEN along with CNL and AC logs are more reliable tools as compared to GR and resistivity logs for interpreting CBM.

### Analyzing the methodological applicability and effectiveness

The employed integrated AI approach provided reliable results in a complex coal-tight sand based geological formation. Q–Q plots provided a basic visual approach to compare rock distributions, but SOMs provide advanced approaches for grouping and mapping high-dimensional data. Q–Q plots and SOM provided distinct methodologies for identifying coal strata, each with its own strengths and limitations. K-means clustering and SOM are clustering approaches and distinguished coal-sand strata based on distinct clusters linked to distinct color codes. Whereas, t-SNE distinguished the coal from sand and shale spatially.

In addition to the applicability of the employed AI methods, their strengths and weaknesses are as follows;

The t-SNE algorithm is proficient in maintaining non-linear associations in the data^70^, which might be advantageous for capturing sophisticated spatial patterns in coal layers. t-SNE often generates distinct clusters in the lower-dimensional space^71^, facilitating the differentiation of various lithological units such as coal layers, sandstones, and shales. t-SNE is a stochastic method, implying that it might provide varying outcomes over multiple runs. This may create unpredictability and complicate the replication of the precise clustering outcomes. t-SNE involves the adjustment of several parameters, including the perplexity parameter, which needs careful tuning. Choosing suitable parameter values may be a difficult task and has the potential to impact the outcomes of the clustering process^72^.

K-means is capable of handling huge datasets and effectively managing a substantial number of data points^73^, making it well-suited for processing vast well logging data or geological images. K-means is a basic clustering technique which is easy to implement and explain^74^. It allocates data points to the closest centroid, that makes it easier to identify the coal layers from other rock types with similar feature patterns. The spherical and equal-sized cluster assumption of K-means is not always applicable to geological data^73^. For instance, coal layers along with sand-shale might provide less-than-ideal clustering findings due to their potentially amorphous forms and inconsistent thicknesses. Identifying the most suitable number of clusters (k) in K-means may be difficult and may need prior geologic knowledge for validation purposes.

Q–Q charts are non-parametric and agnostic to the underlying distribution of data, making them adaptable and suitable for many datasets. Q–Q plots may detect deviations from the anticipated distribution, enabling engineers to find abnormal patterns or outliers^75^, that could correlate to coal strata as well as sand-shale or other geological characteristics. Q–Q plots mainly give qualitative insights into the distributional qualities of data^76^, and may not provide extensive quantitative information on sand-shale and coal layer characteristics or spatial regional linkages.

SOMs maintain the topological links between data points while organizing high-dimensional data into a low-dimensional map helps in visualizing and interpreting the spatial distribution of sand-shale as well as coal strata. SOMs have the capability to detect patterns or clusters within the data^77^, such as coal-sand layers that are spatially distributed, by analyzing similar feature representations in the input space. Particularly for large or high-dimensional datasets, the interpretation of SOM results may prove difficult due to the fact that the relationships between nodes on the map might not correspond explicitly with geological features or lithological boundaries. Particularly for large datasets or high-resolution geological data, training SOMs can be computationally intensive, necessitating substantial processing time and resources^78^.

## Conclusion

The main conclusions are as follows;

1. It is essential to do log normalization and outlier detection prior to a detailed interpretation. Q–Q plot analyzed through normalized AC log suggested three distinct distribution sets. Tight sand and shale show distinct behavior with low to moderate values. However, coal distribution set is distinguished through high AC values.
2. The petrophysical and crossplot analyses showed that GR and resistivity logs are sensitive towards coal with wide range of values, hence making them less reliable to distinguish the lithological variations. Coal is distinguished from the tight sand based on low DEN (1.25 to 1.65 g/cm^3^), low PE (> 1 b/e) high AC (90–150 μs/ft), high CNL (0.45–0.7 dec), comparatively high GR due to Th-bearing minerals, low POR (0–2%), and wide range of medium to high resistivity values. On the other hand, tight sand facies show high DEN (2.5–2.65 g/cm^3^), low-to-medium AC (40–100 μs/ft), moderate PE (2–2.5 b/e), low CNL (0–0.35 dec), low GR (20–60 API), and high POR (8–10%) values. Whereas, shale intercalations are situated within the intermediate spectrum between tight sand and coal.
3. SOM clustered three types of dominating lithofacies in a heterogeneous geological formation of the reservoir unit of about 150 m, each characterized by varying degrees of variability along with color codes. These included tight sands with assigned number 1 (gold color) exhibiting low GR, high DEN and low CNL values suggesting gas-bearing facies. Whereas, coal (gray color, assigned number 2) exhibiting low DEN and high AC as well as CNL values. Shale facies (blue color, assigned number 3) shows moderate ranges indicative of a rock type with a moderate to low potential for gas as compared to tight sand facies, supporting the results of petrophysical and pairplot analyses.
4. The analysis of k-means clustering characterized the rock types into five clusters representing five rock types. Cluster 1 associated good POR values suggesting good-quality tight gas RRT, whereas other clusters 2, 3, 4, and 5 are associated with shale and coal variants exhibiting very low POR values and are termed as poor-quality RRT.
5. Coal was accurately distinguished via t-SNE. Results of coal using t-SNE were used to make a CNL versus DEN crossplot with AC log as magnitude coding that showed the presence of low-rank high-volatile bituminous coal in the study area.
6. In a nutshell, the interpretation approaches employed in this work are of great relevance importance in complex coal-tight sand based geological formations. There are discrepancies between the environments of the various coal strata, therefore a careful attention is needed to interpret the coal layers. Interpreted coal seams can be further assessed for unconventional exploration and development of the CBM deposit.

## Data Availability

The datasets used during the current study available from the corresponding author (Aqsa Anees, aqsaanees@ynu.edu.cn) on reasonable request. But restrictions apply to the availability of these data, which were used under license for the current study, and so are not publicly available.

## Abbreviations

AC: Acoustic log (μs/ft)
DEN: Density log (g/cm^3^)
GR: Gamma ray log (API)
NGR: Natural gamma-ray
CNL: Neutron porosity log (dec)
LLD: Deep resistivity (Ω)
LLS: Shallow resistivity (Ω)
PE: Photoelectric (b/e)
Th: Thorium (ppm)
K: Potassium (%)
U: Uranium (ppm)
POR: Porosity (%)
PERM: Permeability (dec)
Q–Q plot: Quantile–quantile plot
ANN: Artificial neural network
SOM: Self-organizing map
CBM: Coalbed methane
BMU: Best matching units
CO: Carbon monoxide
CO_2_: Carbon dioxide
RRT: Reservoir rock typing
t-SNE: T-Distributed stochastic neighbor embedding
